# Optimizing adaptive stepped-care interventions to change adults’ health behaviors: A systematic review

**DOI:** 10.1017/cts.2023.618

**Published:** 2023-08-25

**Authors:** McKenzie K. Roddy, Angela F. Pfammatter, Lindsay S. Mayberry

**Affiliations:** 1 Department of Medicine, Vanderbilt University Medical Center, Nashville, TN, USA; 2 Center for Health Behavior and Health Education, Vanderbilt University Medical Center, Nashville, TN, USA; 3 College of Education, Health, and Human Sciences, University of Tennessee, Knoxville, TN, USA

**Keywords:** Systematic review, health behavior, adaptive interventions, decision rule, SMART

## Abstract

Chronic diseases are ubiquitous and costly in American populations. Interventions targeting health behavior change to manage chronic diseases are needed, but previous efforts have fallen short of producing meaningful change on average. Adaptive stepped-care interventions, that tailor treatment based on the needs of the individual over time, are a promising new area in health behavior change. We therefore conducted a systematic review of tests of adaptive stepped-care interventions targeting health behavior changes for adults with chronic diseases. We identified 9 completed studies and 13 research protocols testing adaptive stepped-care interventions for health behavior change. The most common health behaviors targeted were substance use, weight management, and smoking cessation. All identified studies test intermediary tailoring for treatment non-responders via sequential multiple assignment randomized trials (SMARTs) or singly randomized trials (SRTs); none test baseline tailoring. From completed studies, there were few differences between embedded adaptive interventions and minimal differences between those classified as treatment responders and non-responders. In conclusion, updates to this work will be needed as protocols identified here publish results. Future research could explore baseline tailoring variables, apply methods to additional health behaviors and target populations, test tapering interventions for treatment responders, and consider adults’ context when adapting interventions.

## Introduction

Chronic diseases, noncommunicable diseases that last at least a year and require ongoing medical attention (e.g., cancer, diabetes, obesity, and heart disease), are the leading cause of death and disability as well as being responsible for the largest proportion of US healthcare costs [1]. Furthermore, in 2019, more than half of Americans between the ages of 18 and 34 reported having at least one chronic disease [2], highlighting that the burden of chronic diseases will continue to grow as the population ages. Many chronic diseases are caused or exacerbated by health behaviors including tobacco use, poor nutrition, and physical inactivity [1]. Interventions targeting health behavior change are well positioned to address these growing problems in the population, and numerous such interventions exist.

However, health behavior change interventions have small or null effects on average. A review of internet delivered interventions to promote health behavior change reported a small average effect (*d* = 0.16, 95% CI 0.09–0.23) [3]. Within specific disease contexts, research has found response to weight loss interventions is suboptimal [4] and mixed results for improving hemoglobin A1c (HbA1c) for adults with diabetes [5,6]. Further, response to treatment is often heterogeneous across individuals. For example, research shows adults with elevated baseline HbA1c have greater reductions in HbA1c during a self-care support intervention than adults with moderately elevated baseline HbA1c.[7] Finally, many chronic conditions wax and wane over time and the intervention needs of individuals are therefore not static [8]. With this context in mind, there have been calls for further research and applications of adaptive interventions in the management of chronic diseases [9,10].

Adaptive interventions are defined by a sequence of decision rules or if-then statements that specify whether, when, and how to alter the intervention [8,10]. For example, an adaptive intervention for weight loss might specify that all participants receive diet and exercise counseling for 8 weeks. At 8 weeks, if participants have not lost>5lbs, they are referred to group sessions for an additional 6 weeks. However, if at 8 weeks participants have lost ≥ 5lbs, they continue with their current treatment plan. As in this example, decision rules for adaptive interventions are centered on characteristics of the individual such as level of the outcome measured at baseline or progress towards the outcome measured during the intervention [8]. Clinical judgment and prior research inform the timing of when decision rules are applied, as well as the decision rules themselves, and what adaptations are applied [10]. Adaptive interventions are well suited to heterogeneous populations, populations with heterogeneous treatment responses, and/or conditions with high frequency of relapse or waxing and waning of symptoms. Benefits of adaptive interventions include reducing wasted resources, increasing compliance, reducing negative effects associated with inappropriate treatment for certain individuals, and enhancing potency [10].

Broadly, there are two classes of adaptive interventions: dynamic and stepped care. Dynamic interventions, including just-in-time adaptive interventions (JITAI), tailor the intervention daily or hourly in response to the state of the individual which frequently changes (e.g., urges to smoke). Stepped care interventions tailor the intervention less often, based on individuals’ responses to the intervention (e.g., weight loss). This review will focus on stepped care adaptive interventions, where care is viewed as a continuum through which individuals can move – increasing treatment for nonresponders and tapering treatment as individuals progress over weeks or months.

Clinical trials of stepped-care adaptive interventions are designed to test the decision rules that form the backbone of the final intervention. If an adaptive intervention is developed using prior literature and clinical judgment only to inform the decision rules, the specific decision rules cannot be empirically tested. Rather, adaptive interventions iteratively developed by creating and then testing decision rules serve to produce the most knowledge efficiently. The aspects of decision rules to be tested include the baseline tailoring variables and the intermediary tailoring variables to be used (if any) and at what levels. Aspects of a decision rule to be tested could also include when to tailor the intervention and/or what type of treatment or modality of treatment delivery should be used in different cases. Trials of adaptive interventions can test the efficacy of multiple embedded interventions [11]. They often include options for further exploratory analyses beyond the main research questions, though, similar to other experimental designs, power may be a concern due to small sample sizes in some cells (e.g., when testing moderation of treatment effects).

A Sequential Multiple Assignment Randomized Trial (SMART) is a research design developed for testing adaptive interventions systematically and efficiently. SMARTs are inherently resource efficient, asking multiple questions about the components of an adaptive intervention in a high-quality manner without unduly increasing sample size [9]. Further, adaptive interventions tested via SMARTs mirror clinical practice in that individuals’ unique needs and circumstances inform the treatment delivered [12]. Importantly, when designed to systematically test decision rules, adaptive interventions are replicable in ways that traditional clinical practice is not [10]. Due to their multiple randomization points, SMARTs are especially well suited to test the rules that govern decisions made in stepped-care adaptive interventions.

### Current Study

Rigorous tests optimizing adaptive interventions are relatively new experimental designs that are being applied to health behavior interventions. Interventions for health behavior change have historically struggled to, on average, produce clinically meaningful change and maintain results, providing opportunity for adaptive interventions to improve outcomes. Within the field of health behavior interventions, there are often target behaviors (e.g., increasing physical activity), which can impact several outcomes of interest (e.g., weight management, glycemic control). Additionally, there are target behaviors (e.g., medication adherence) that are applicable in multiple contexts (e.g., type 2 diabetes, HIV prevention and treatment). Given the emerging state of these methods in this field and the commonality of behaviors targeted throughout the field, we seek to systematically review applications of optimization methods to adaptive intervention development. Specifically, we are interested in optimization of adaptive interventions for adults to change health behaviors to learn how tailoring is being done and tested. Results may help highlight the types of questions being asked about adaptive interventions and inform future trial designs.

## Materials and Methods

### Data Sources

Our systematic review covered studies published up to December 2022. The investigators searched Google Scholar for eligible studies using a combination of key words (sequential multiple assignment randomized trial, SMART, adaptive, optimize AND health behaviors, behavior change, alcohol, and drug). We also systematically searched the reference lists of included studies and recent reviews of similar topics (e.g., Bigirumurame *et al*. [13], Jaehne *et al*. [14], and Miller [15]), as well as searched for papers that cite key methodological references (e.g., Murphy [16] and Collins *et al*. [9])

### Study Selection and Data Extraction

We reviewed titles and abstracts of citations and identified eligible articles. Inclusion criteria were studies that (1) seek to optimize adaptive stepped-care interventions, (2) target an observable health behavior change as proximal or distal outcome, (3) test one or more decision rules, (4) are systematically different across individuals and/or across time based on participant characteristics or clinical characteristic (vs. fixed intervention, where all participants in a condition receive identical treatment), (5) included adults in the sample, and 6) were published in English.

We included both protocol papers and articles with results from such studies. Investigators collected the following information from each article that was eligible: description of the sample, research design, initial treatment, duration to tailoring, definition of treatment non-response, subsequent treatments and tailoring, and outcomes when available or decisions to be tested for protocols.

**Figure 1.**
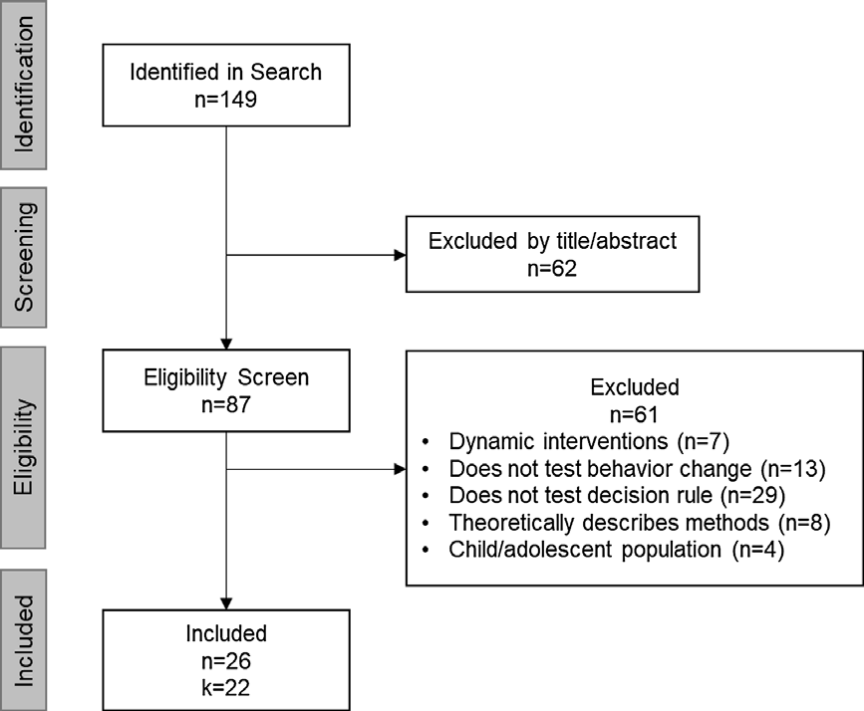
Systematic review inclusion flow diagram.

Literature searches yielded 149 articles. The first author read the titles and abstracts of these articles to determine if they were appropriate for initial inclusion, resulting in 87 articles. Of these, 61 articles were excluded because they described studies testing dynamic interventions (e.g., JITAI or micro randomized trials; *n* = 7), did not evaluate health behavior change (*n* = 13), did not test decision rules (*n* = 29), theoretically described methods only (*n* = 8), or studied exclusively child/adolescent populations (*n* = 4). The resulting sample yielded *n* = 26 studies describing *k* = 22 unique research projects, see Fig. 1 for flow diagram of inclusion. If a study had published results and a published protocol, or multiple papers describing results, they were grouped together for description below.

Given the diversity of health behaviors targeted and methods employed, we used a narrative synthesis approach to describe and identify patterns of tests of decision rules across included studies.[17] We provide results overall and also separately, studies reporting outcomes of trials and studies reporting protocols.

## Results

Of the 22 included research projects, 9 tested decision rules for adaptive stepped-care interventions and 13 were research protocols (See Table 1). All studies were optimization studies; however, one compared a control condition to a SMART [18]. Almost all studies (25/26) were published in the last 18 years since 2005. All included projects utilized Sequential Multiple Assignment Randomized Trials (SMARTs) or singly randomized trials (SRTs; an experimental design where participants are randomized once over the course of treatment – here mid-way through treatment) [19]. Every project but one [20] tested intermediary tailoring for treatment nonresponders based on progress towards the primary outcome. No studies evaluated change in hypothesized behavioral mediators as indicative of nonresponse to the intervention to inform tailoring. Intermediary tailoring occurred between 2 and 14 weeks. Only three protocols tested stepping down or tapering care for treatment responders [21–23], while no completed studies tested reducing care for responders. No identified projects tested baseline tailoring. Two studies randomized the length of time to tailoring or initial intervention [23,24]. No participants received an intervention with the first stage tailored to their presenting criteria (e.g., baseline disease severity).

**Table 1.** Characteristics of included studies (k = 22)

Stage of work	
Completed research	9
Protocol	13
Experimental design	
Single randomized trial (SRT)	5
Sequential multiple assignment randomized trial (SMART)	17
Health behavior target	
Substance use	6
Weight management	5
Smoking cessation	4
Medication adherence	3
Physical activity	3
Glycemic management	1
*Among Completed Research (k = 9)*	
Gender of participants	
<30% female	1
30–70% female	6
>70% female	2
Race/ethnicity of participants	
>25% racial/ethnic minority	2
≤ 25% racial/ethnic minority	5
Not reported	2
Sample size	
≤ 50	2
51–150	3
151–400	0
401–900	4

Several projects targeted substance use (*k* = 6) [18,25–31]. weight management (*k* = 5) [20,24,32–35], and smoking cessation (*k* = 4) [23,36–38]; fewer targeted medication adherence (*k* = 3) [21,22,39,40], physical activity (*k* = 3) [41–43], and glycemic management (*k* = 1) [44]. The projects targeting medication adherence were focused on antiretroviral therapy (ART) for human immunodeficiency virus (HIV) [21,22,39] and pre-exposure prophylaxis (PrEP) for HIV prevention [40]. Of note, alcohol use studies were mostly tested in college populations with an aim of prevention following early warning signs [25,26,28]. Some smoking cessation and cocaine use projects tested combined behavioral and pharmaceutical stepped-care interventions [31,38].

### Completed Research

***Completed Research Design.*** Of the 9 projects, 5 were SRTs and 4 were SMARTs. The median [25^th^, 75^th^ interquartile range (IQR)] sample size of completed research was 136 [55, 500]. All SRTs provided the same intervention to all participants initially and randomized nonresponders to subsequent intervention elements (Table 2). In all completed studies, responders continued the first line of treatment or moved to assessment only but were not randomly assigned. All SMARTs tested personalization[18,41] and duration to tailoring[30,33] in the first randomization and additional resources or swapping to the alternate condition during subsequent tailoring for non-responders.

**Table 2.** Description of included studies

Authors and year	Sample and design	Initial treatment	Duration to tailoring	Definition of nonresponse	Subsequent treatment	Outcomes / decisions tested
	*Completed research testing decision rules for adaptive stepped-care interventions (k = 9)*					
Borsari *et al*., 2007 [25]	43 college students referred to alcohol counseling in SRT	11-min brief intervention to reduce drinking administered by peer counselors including informational booklet	4 weeks	3 or more binge drinking episodes in past 30 days and/or >= 2 self-endorsed alcohol-related problems	Responders: assessment-only Nonresponders: rerandomized to brief motivational intervention or assessment-only control	Among heavy drinkers, within group effect sizes suggest small to moderate reductions in drinking and alcohol-related problems for the brief motivational group at 2.5 months post-baseline. No findings for responders and no group differences for nonresponders.
Bosari *et al*., 2012 [26]	598 college students mandated to attend alcohol program in SRT	15-min brief advice session to reduce drinking including informational booklet	6 weeks	4 or more heavy drinking episodes and/or >= 5 self-endorsed consequence of alcohol	Responders: discontinue treatment – assessment only Nonresponders: rerandomized to brief motivational intervention or assessment-only control	Among heavy drinkers, assignment to brief motivational intervention reduced number of alcohol-related problems compared to those who received assessment only over 9 months. Low-risk drinkers show stable alcohol use over 9 months follow-up
Breslin *et al*., 1998 [27]	136 adults with problematic drinking in SRT	All participants received 4 weekly outpatient individual sessions plus two phone contacts based on motivational approaches	12 weeks	>12 drinks per week during first three treatment sessions	Responders: assessment only Nonresponders: randomized to standard program or standard program plus memory and motivation supplemental intervention	Responders had higher percent days abstinent than either nonresponder group at 6 months. No differences between nonresponder groups.
Carels *et al*., 2008 [32]	54 overweight or obese adults in SRT	Randomized to self-help or therapist-assisted self-help	14 weeks	<5% weight loss	Responders: assessment only Nonresponders: initiate 3-month group-delivered behavioral weight loss program	Weight loss not improved by addition of therapist-assistance during first stage of treatment. Addition of group behavioral weight loss program for nonresponders did not improve weight loss at 6.5 months.
Carels *et al*., 2019{Carels, 2019 #156}	53 overweight or obese adults in SRT	All participants received a self-help version of the DPP with technology-assisted exercise and caloric monitoring via technology	8 weeks	<2.5% weight loss	Responders: continue Nonresponders: randomized to continue with self-help or add weekly acceptance-based weight loss group	Responders lost on average 5.3% of weight over 16-week intervention; nonresponders assigned to self-help lost 0.4% of weight and nonresponders assigned to acceptance group lost 1.3% of weight.
Gonze *et al*., 2020 [41]	18 adults with insufficient physical activity in feasibility SMART	Randomized to app or app plus tailored messages or control	12 weeks	Nonpositive slope in linear relationship between weeks and steps per day	Responders: continued first-stage treatment Nonresponders: rerandomized to whichever condition they didn't have first or app plus tailored messages plus gamification	No differences in steps per day between interventions and control at 6 months. Rerandomization of app nonresponders led to more steps per day, but no differences between the second stage treatments at 6 months.
Lynden *et al*., 2022 [28]; Patrick *et al*., 2020 [29]; Patrick *et al*., 2021 [18]	891 first year full time college students aged 18-21 in SMART vs control	Randomized to personalized normative feedback plus self-monitoring prior first semester or during first month of first semester	2, 4, 6, or 8 weeks – whichever first identified nonresponse	4/5+ drinks for women/men in 2-hour period	Responders: assessment-only Nonresponders: rerandomized to resource email or online health coach	Adaptive preventative intervention was not superior to control in reducing drinking at 9 months. No differences among embedded adaptive preventative interventions. Among nonresponders, resource email vs health coach led to greater health services utilization at 9 months.
McKay *et al*., 2015 [30]	500 adults with alcohol- and/or cocaine-dependence in SMART	Intensive outpatient program with 9 hours group treatment/week	2 weeks and/or 3–8 weeks	Not engaged in intensive outpatient program at 2 weeks or dropped out between 3 and8 weeks	Responders: continue intensive outpatient program Nonresponders at 2 weeks OR dropped out 3-8 weeks: rerandomized for motivational interviewing calls focused on engagement in intensive outpatient program or motivational interviewing calls focused on patient choice of treatment Nonresponders at 2 weeks THEN dropped out 3-8 weeks: re-randomized to motivational interviewing calls focused on patient choice or no further care	For adults with alcohol dependence, motivational interviewing for intensive outpatient were less likely to have months with drinking days and months with heavy drinking days than patient choice at 6 months. There were no differences in cocaine use for adults with cocaine dependence.
Sherwood *et al*., 2016 [24]; Sherwood *et al*., 2022 [33]	468 adults aged 21–70 with BMI 30–45 kg/m^2^ in SMART	Behavioral weight loss treatment	Randomized to 3 or 7 weeks	<=2.5% weight loss at 3 weeks or <= 5% weight loss at 7 weeks	Responders: continue first-stage treatment Nonresponders: re-randomized to augment first phase treatment with portion-controlled meals or switch to acceptance-based treatment	Among nonresponders, portion-controlled meals participants and acceptance-based treatment participants had similar weight loss at 18 months. Similar weight loss between 3- and 7-week assessment points; however, 3-week portion-controlled meal participants lost more weight than 7-week portion-controlled meal participants.
	*Protocols Testing Decision Rules for Adaptive Stepped-Care Interventions (k = 13)*					
Belzer *et al*., 2018 [21], Naar *et al*., 2019 [22]	Planned enrollment 190 youth living with HIV aged 15-24 in SMART	Randomized to phone call support or text messages for medication adherence	3 months	Viral load >= 200 copies/mL	Responders: If phone call support re-randomized to tapered phone call support or standard care. If text messages, rerandomized to tapered text messages or standard care. Nonresponders: Rerandomized to incentivized cell phone support or incentivized text messages	Outcome will be viral load suppression rate at 3 months Phone call support vs text messages as first-line treatment Incentivized phone call vs incentivized texts messages for nonresponders at 6 months Tapered vs standard care for responders at 6 months
Buchholz *et al*., 2020 [42]	Planned enrollment 312 inactive women aged 18–70 in SMART	Randomized to enhanced physical activity monitor or enhanced physical activity monitor plus text messages	8 weeks	Missing data or average steps do not exceed the short-term goal of 600 steps above baseline average for two of the three weeks in weeks 6–8	Responders: continue first-stage treatment Nonresponders: continue first-stage treatment plus rerandomized to personal calls or group meetings for subsequent 6 months	Outcomes will be physical activity, aerobic fitness, BMI, and waist circumference at 8 and 12 months Benefit of adding texts to enhanced physical activity monitor as first-line treatment Adding personalized calls vs group meetings for nonresponders
Comins *et al*., 2019 [39]	Planned enrollment 800 female sex workers living with HIV in SMART	Randomized to decentralized treatment program or individualized case management	6 months	>50 copies/ml viral load	Responders: Randomized to standard of care or continue first- stage treatment Nonresponders: Randomized to continue first-stage treatment or continue first-stage treatment plus other first-stage treatment (combination)	Outcomes will be viral load at 18 months Decentralized treatment program vs individualized case management as first-line treatment Combined treatment vs continue at 6 months for nonresponders Standard of care vs continue at 6 months for responders
Corbin *et al*., 2022 [44]	68 young adults with type 1 diabetes, BMI 27–39.9 kg/m^2^, and aged 19–30 in SMART	Randomized to one of three diets. All received 2 60-minute in-person sessions and 5–10 minute phone check-ins with registered dietician	3 and 6 months (twice during study)	Participant reported diet unacceptable, not achieving 2% weight loss since previous visit, and/or HbA1c increased >= 0.5% and problematic hypoglycemia	Responders: continue with diet from previous phase, including 2 60-in in-person sessions with registered dietician and 3 10-minute phone check-ins Nonresponders: rerandomized to one of remaining untested study diets including 2 60-minute in-person sessions with registered dietician and 3 10-mintue phone check-ins	Outcomes will be weight change and glycemic management (HbA1c) at 3 months Three diets as first-line treatment (not powered to test second or third diets) at 6 and 9 months
Edelman *et al*., 2021 [36]	Planned enrollment of 538 adults living with HIV who smoke tobacco in SMART	Randomized to nicotine replacement therapy with or without contingency management	12 weeks	Smoking in 7 days prior to assessment	Responders: continue first-stage treatment Nonresponders: if in contingency group, re-randomized to enhanced contingency management or prescription medication. If in nicotine replacement only group rerandomized to contingency management or prescription medication	Outcomes will be smoking status and HIV-related outcomes at 6 months Benefit of adding contingency management to nicotine replacement therapy as first-line treatment Contingency management vs prescription medication for nonresponders
Fernandez *et al*., 2020 [37]	Planned enrollment 33 clinics, 6000 tobacco users in cluster randomized SMART	Clinics randomized to opt-in or opt-out strategy. All participants receive same initial treatment asking about smoking, advice to quit, and connected to resources	2 weeks and 6 months	Not enrolled in state quit line	*At participant level:* Responders: continue prior stage treatment Nonresponders: at 2 weeks rerandomized to treatment as usual or text messages; at 6 months in text message condition rerandomized to text messages or text messages plus motivation and problem solving	Outcomes will be state quit line enrollment and abstinence at 12 months Opt-in vs opt-out at clinic level Benefit of adding texts for nonresponders Benefit of adding motivation and problem solving for nonresponders to text messages
Fu *et al*., 2017 [23]	Planned enrollment 1000 current daily smokers at high risk for lung cancer who are willing to quit smoking in SMART	Seven counseling calls over 8 weeks covering problem solving, skills training, social support, nicotine replacement use, and relapse prevention	Randomized to 4 or 8 weeks	Any smoking within 7 days prior to assessment or unable to assess	Responders: rerandomized to continue monthly contact or decrease to quarterly contact Nonresponders: rerandomized to continue monthly contact or continue treatment plus medication therapy management	Outcome will be 6-month prolonged abstinence from smoking 18 months post randomization 4- vs 8-week duration to assess nonresponse Monthly vs quarterly contact for responders Monthly contact vs medication therapy management for nonresponders
Germeroth *et al*., 2019 [20]	Planned enrollment 300 pregnant persons with prepregnancy BMI≥25 kg/m^2^ in SMART	Randomized at start of prenatal care to intervention for weight and healthy behaviors or treatment as usual	Prior to delivery of pregnancy	n/a	Randomized at end of pregnancy to intervention for weight and healthy behaviors or treatment as usual	Primary outcome will be weight attend of pregnancy, 6 and 12 months postpartum Treatment as usual vs intervention during pregnancy Treatment as usual vs intervention during postpartum Benefit of continuing intervention to postpartum vs treatment as usual postpartum
Pfammatter *et al*., 2019 [34]	Planned enrollment 400 adults aged 18–60 years with BMI 27–45 kg/m^2^ in SMART	Randomized to app or app plus coaching	2, 4, or 8 weeks, whichever first identified nonresponse	< .5lbs weight loss per week on average	Responders: continue first-stage treatment Nonresponders: rerandomized to add text messages or add text messages, coaching (if not initially assigned), and meal replacements	Outcomes will be change in weight at 6 months Benefit of adding coach to app as first-line treatment Benefit of adding text messages vs text messages plus coaching and meal replacements for nonresponders
Schmitz *et al*., 2018 [31]	Planned enrollment of 160 adults with cocaine use disorder of at least moderate severity in SMART	Randomized to acceptance and commitment therapy plus contingency management or drug counseling plus contingency management	4 weeks	Did not submit 6 consecutive (3 per week for 2 weeks) clean urine samples	Responders: continue first-stage treatment Nonresponders: continue first-phase treatment and rerandomized to add placebo or medication to reduce cocaine use	Outcome will be cocaine abstinence at 3 months Acceptance and commitment therapy vs drug counseling as first line treatment Benefit of adding medication vs continue first-line treatment for nonresponders
Simões, *et al*., 2019 [43]	42 adults aged 20+	Randomized to app plus tailored messages; app, tailored messages, and gamification, or physical activity counseling (control)	6 weeks	Zero or negative slope of daily step counts during first 6 weeks	Responders: continue first- stage treatment Nonresponders: Rerandomized to alternate first-stage treatment or app, tailored messages, and enhanced gamification	Average number of daily steps at 3 and 6 months
Velloza *et al*., 2022 [40]	Planned enrollment 500 women aged 18–25 years who initiate PrEP for HIV prevention in SMART	Randomized to counseling plus texts or counseling plus support groups	3 months	Insufficient level of PrEP in blood sample or missed 3-month study visit	Responders: continue first-stage treatment Nonresponders: continue first-stage treatment and rerandomized to quarterly drug-level feedback or monthly issue-focused counseling	Outcome is adherence to PrEP via blood sample at 9 months Text messages vs support groups as first- line treatment Quarterly drug-level feedback vs monthly issue-focused counseling for nonresponders
Zhao *et al*., 2022 [38]	Planned enrollment 1200 daily cigarette smokers in SMART	Randomized to personalized instant messaging or regular instant messaging. All participants receive brief cessation advice plus referral assistance to smoking cessation services	1 month	Smoking in 7 days prior to assessment	Responders: continue first phase treatment Nonresponders: If personalized messages, rerandomized to continue first-stage treatment or optional combined interventions (multimedia messages, nicotine replacement therapy sampling, financial incentive for active referral, phone counseling, family/peer support group chat). If regular messages, rerandomized to continue first-stage treatment or personalized instant messages.	Outcome will be nicotine abstinence at 3 and 6 months Personalized vs regular instant messaging as first-line treatment Benefit of adding combined intervention for personalized messaging nonresponders Benefit of personalized messaging for regular messaging nonresponders

HIV = human immunodeficiency virus; SMART = sequential multiple assignment randomized trial; BMI = body mass index; HbA1c = hemoglobin A1c; PrEP = preexposure prophylaxis; RCT = randomized control trial; SRT = single randomized trial.

***Completed Research Context and Findings.*** Completed research has tested decision rules for health behaviors including substance use [18,25–27,30], weight loss [32,33,35,45], and physical activity [41]. In general, there were few differences between embedded adaptive interventions in the completed research studies, meaning there were not differential effects found for various treatments or modalities tested. As no completed studies tested tapering, we are unable to draw conclusions about the appropriateness of discontinuing treatment for responders. Furthermore, most studies did not report finding differences among treatment nonresponders who were rerandomized, meaning providing additional or different treatment elements to nonresponders did not change their trajectories [18,25,27,32,33,35,41,45]. For example, the addition of brief motivational interviewing for nonresponders to brief advice sessions for alcohol misuse did not reduce alcohol consumption [25,26]. Therapist-assisted self-help did not outperform self-help, and adding group-delivered behavioral weight loss programing for non-responders did not improve weight loss [32]. Delivering an alcohol prevention program prior to or during the first semester had similar outcomes [18]. However, there were two exceptions: Bosari and colleagues did not find differences on their main outcome of alcohol consumption but did see an effect for reducing number of alcohol-related problems for nonresponders who were randomized to receive additional treatment components [26]. Additionally, McKay and colleagues found motivational interviewing for intensive outpatient treatment had better outcomes than motivational interviewing for patient choice[30].

### Protocols

***Protocol Design.*** All 13 of the protocols described herein employ SMARTs to optimize adaptive interventions, randomizing at least twice during the intervention (Table 2). The median [IQR] sample size of protocols is 400 [190, 800]. Protocols are posing questions during the first phase of treatment regarding modality of delivery or theoretical orientation [21,31,39,40,43], benefit of adding treatment to standard care [20,34,36,38,42], implementation strategies [37], or duration of time to tailoring [23]. Additionally, most studies are using subsequent randomization for treatment nonresponders. In an effort to enhance effects for treatment non-responders, protocols are testing strategies to include adding incentives [21], adding human-delivered elements such as calls or counseling [34,36,37,42], adding prescription medication [31,36,38], adding texts [34,37,38], adding medication counseling or feedback [23,40], combining treatments [39], adding peer elements [43], and adding custom diets [34,44]. For example, Bucholz and colleagues [42] are enrolling 312 inactive women in a SMART aimed to increase physical activity. Initially, all women are provided an enhanced physical activity monitor and are randomized to additionally receive text messages or not. At 8 weeks, women who do not exceed the target step count or had missing data are rerandomized to add personal calls or group meetings while continuing their first-stage treatment. Women who respond to the first stage of treatment as indicated by meeting or exceeding the targe step count continued with their initial treatment assignment.

There are three notable exceptions to the common approach taken by protocols described above. Fernandez and colleagues are cluster randomizing wherein the first-stage clinics are randomized to opt-in or opt-out implementation strategies and patients are randomized at the second stage based on treatment response [37]. Corbin and colleagues randomize participants to one of three diets and rerandomize treatment nonresponders to unassigned diets resulting in participants being exposed to between one and three diets depending on treatment response [44]. Finally, Germeroth and colleagues are using a design where everyone is rerandomized, regardless of response status, such that some individuals remain with their original treatment assignment and others swap [20].

Finally, in addition to testing adaptations for treatment nonresponders, Belzer and colleagues [21], Fu and colleagues [23], and Comins and colleagues [39] are testing adaptations for treatment responders including tapering vs standard care, decreasing frequency of care, and continuing treatment as assigned vs standard care, respectively.

***Protocol Context.*** Protocols are set to test a variety of health behaviors including smoking cessation [23,36–38], medication adherence [21,39,40], physical activity [42,43], glycemic management [44], weight management [20,34], and cocaine abstinence [31]. All protocols are set in outpatient or community settings.

## Discussion

Adaptive interventions for health behavior change are a developing area of science. All (but one study) were published in the last 18 years (since 2005) and the majority (73%; 19/26) were published in the last 5 years (since 2018). Optimization methods for adaptive interventions have recently gained traction. Given the time needed to procure grant funding and publish results, more research in this area is expected soon. Indeed, this review found more studies as protocols than completed work, highlighting the infancy of this field and the promise of more information coming. Of note, SRTs were present in completed studies but not in protocols, likely due to the availability of a more novel trial design (SMART) better suited to answer questions about adaptive interventions with repeated opportunities for randomization. It is therefore likely that uptake of these methods will continue to increase. Our review can inform this uptake; therefore, we make the following commentary on design and contextual considerations.

### Design Considerations

Intermediary tailoring was ubiquitous among reviewed studies, mostly focusing on treatment nonresponse defined by progress toward the primary outcome. Models of adaptive interventions have suggested using known treatment mediators or proximal outcomes to inform intermediary tailoring [10]. It is possible these approaches are not being utilized as strong treatment mediators are difficult to identify, or because health behaviors themselves are often the proximal outcomes in relation to more distal outcomes of clinical indicators, morbidity, and mortality. One study used engagement as the intermediary tailoring variable [37], and this approach could likewise be used in future trials.

A minority of studies are systematically testing intervention reductions for treatment responders. These questions have important implications for the cost-effectiveness of interventions, namely providing enough resources to achieve the desired outcomes without providing additional unnecessary resources. Continuing to test tapering or stepping down care for treatment responders is an important question to consider in future work.

There is a paucity of research testing baseline tailoring variables. In the future, it may be useful to explore tailoring at baseline where individuals are receiving a first-stage treatment that is likely to be effective for them. The literature can help to generate hypotheses about which treatment may be best for a particular individual or subgroup. Variables that moderate fixed interventions may be informative for identifying baseline tailoring variables to test. However, it may be that a cluster or composite of variables create a behavioral phenotype (rather than a single variable) that would be predictive of treatment success. In fact, there is emerging work identifying associations between clusters of lifestyle risk factors and overweight/obesity [46] as well as typologies of family functioning related to depressive symptoms for patients with heart failure [47] and self-management and psychosocial well-being for adults with type 2 diabetes [48]. An interesting future direction for this work would test these typologies or behavioral phenotypes as predictors of treatment outcome and potentially used as tailoring variables in the future.

One novel study described here by Fernandez and colleagues is combining implementation methods with adaptive methods [37]. Developing stepped care interventions that are ready to be implemented holds promise, and future work in adaptive methods could contain more implementation methods and measures in order to ready these interventions for uptake in the real world.

### Contextual Considerations

The most frequent health behaviors to be tested in optimizing adaptive methods were substance use, weight management, and tobacco cessation. It is logical these were some of the first health behaviors targeted by these methods due to modest effect sizes [4,49] and high rates of relapse [50]. In fact, a prior systematic review on stepped care interventions for alcohol and tobacco cessation identified a number of interventions being tested [14]. Likewise, a previous review of adaptive interventions (including JITAI) for weight loss or sedentary behavior identified eight adaptive interventions which showed initial promise for JITAI to reduce sedentary behaviors [15], but to our knowledge these studies largely have not been replicated. More recent efforts include these domains as well as expanding into medication adherence, physical activity, glycemic management, and cocaine abstinence. More research is needed in these and other areas. Adaptive interventions for medication adherence are currently only being tested with adults with HIV and in the prevention of HIV (PrEP) [21,39,40]. Opportunities for medication adherence interventions for adults with chronic diseases such as type 2 diabetes, heart failure, or chronic kidney disease could benefit from their learnings and further these methods. Additionally, interventions that target multiple self-management behaviors such as medication adherence as well as healthy diet, exercise, and stress management could be applicable to multiple chronic disease contexts. There is opportunity to test an adaptive, trans-diagnostic self-management intervention across multiple disease contexts.

In addition to continuing to explore individual variables to use as baseline and intermediary tailoring variables, optimizing adaptive interventions could investigate factors external to the individual to inform optimization of adaptive interventions. Understanding the larger context in which adults are making health behavior changes could further match interventions to individuals in order to maximize effects and enhance engagement. For example, the social context in which adults are making behavior changes could inform which type of intervention is offered (i.e., individual or family). Alternatively, the environment in which adults are making behavior change (i.e., proximity to community health centers) could inform the delivery modality (i.e., in-person or telehealth). Health behavior changes occur in the daily lives and environments of adults seeking to implement them. Finally, all studies reviewed here occurred in the outpatient or community setting. Given the ability of adaptive interventions to change the course of treatment over time, there is opportunity to recruit participants and/or test adaptive interventions in-patient as well as through transitions of care.

### Early Findings Considerations

There are a few key learnings from the findings of completed research studies. First, most did not find differential benefit from adaptations tested. Health behavior change is difficult historically, and that trend continues here. However, the number of decision rules available to be tested is nearly limitless. More work is needed to understand what adaptations are needed for whom. Second, the samples in which these studies were conducted were majority non-Hispanic white. More diversity is needed in the samples used to develop adaptive interventions. Third, several completed research studies had small or moderate sample sizes. Finally, there may be characteristics of individuals that inform intervention readiness or motivation that could be used as baseline tailoring variables to match intervention to person and tailor interventions. Continued intervention optimization could untangle these questions.

### Limitations and Future Directions

Despite the thorough search strategy used, some work optimizing adaptive interventions for health behavior change may have not been identified and omitted from this review. Specifically, we did not search for dissertations or other unpublished documents on this topic. Given the early stage at which this area of research is currently situated, future work will be needed to update the results described here. Due to the wide variety of health behaviors targeted, variety of decision rules tested, and limited number of studies with results, we were unable to complete a meta-analysis. No studies employed factorial designs when testing aspects of decision rules; however, this presents an area of opportunity in the future.

Studies were limited to using two research trial designs: the SRT and the SMART. While SMART lends itself well to testing specific questions regarding the decision rules used to adapt an intervention, other trial designs could be a better fit depending on the research question. For example, if multiple intervention components need testing alongside a single point of adaptation, a factorial trial might be a better option. For example, a factorial trial can answer questions about an adaptation such as adding a component or not after a time of nonresponse while simultaneously testing components to add to a constant intervention component. In this situation, one of the factors to be tested could be adding a coach at the midpoint for nonresponders. This factor could have two levels (yes/no) and groups in the “yes” level would add a coach for nonresponders at the midpoint, while groups in the “no” level would not. Additionally, there are emerging methods for hybrid experimental designs – combining stepped care adaptations with dynamic adaptations for interventions that adapt at multiple timescales [11]. As health behavior intervention science progresses, we expect that more designs will be used to optimize interventions in various ways.

Despite these limitations, to our knowledge this is the first systematic review to highlight the application of optimizing adaptive interventions for health behavior change, broadly. Given the nascency of the field, looking across health behaviors rather than focus on a particular behavior or disease context allows us to learn from a variety of well-designed studies.

## Conclusions

Behavior change interventions have had small average effects historically [3]. These novel designs can help us test adaptations of treatments to be a better fit across the board thereby increasing effects and having positive outcomes for more of the population. Additionally, focusing efforts on improving effects for individuals who have not benefited from interventions historically serves to improve the equity of this work as well as boost the overall or average effect sizes of interventions. Methods for optimizing adaptive interventions are being applied to interventions for health behavior change. There are several published examples and more protocols that will soon have results to add to the field. Opportunities for future research are abundant including identifying and testing baseline tailoring variables, applying the methods to additional health behaviors and target populations, rigorous testing of tapering interventions for treatment responders, and considering adults’ context or modality of delivery when adapting interventions.

## References

[1] NCCDPHP. National Center for Chronic Disease Prevention and Health Promotion (NCCDPHP). Atlanta, GA. https://www.cdc.gov/chronicdisease/about/index.htm#print.

[2] Watson KB , Carlson SA , Loustalot F , et al. Chronic conditions among adults aged 18–34 years—United States, 2019. Morb Mortal Wkly Rep. 2022;71(30):964–970. 10.15585/mmwr.mm7130a3 PMC9345173 35900929

[3] Webb T , Joseph J , Yardley L , Michie S. Using the internet to promote health behavior change: a systematic review and meta-analysis of the impact of theoretical basis, use of behavior change techniques, and mode of delivery on efficacy. J Med Internet Res. 2010;12(1):e1376. 10.2196/jmir.1376 PMC2836773 20164043

[4] Franz MJ , VanWormer JJ , Crain AL , et al. Weight-loss outcomes: a systematic review and meta-analysis of weight-loss clinical trials with a minimum 1-year follow-up. J Am Diet Assoc. 2007;107(10):1755–1767. 17904936 10.1016/j.jada.2007.07.017

[5] Greenwood DA , Gee PM , Fatkin KJ , Peeples M. A systematic review of reviews evaluating technology-enabled diabetes self-management education and support. J diabetes Sci Technol. 2017;11(5):1015–1027. 28560898 10.1177/1932296817713506 PMC5951000

[6] Fitzpatrick SL , Schumann KP , Hill-Briggs F. Problem solving interventions for diabetes self-management and control: a systematic review of the literature. Diabetes Res Clin Pract. 2013;100(2):145–161. 23312614 10.1016/j.diabres.2012.12.016 PMC3633671

[7] Chrvala CA , Sherr D , Lipman RD. Diabetes self-management education for adults with type 2 diabetes mellitus: a systematic review of the effect on glycemic control. Patient Educ Couns. 2016;99(6):926–943. 26658704 10.1016/j.pec.2015.11.003

[8] Almirall D , Nahum-Shani I , Sherwood NE , Murphy SA. Introduction to SMART designs for the development of adaptive interventions: with application to weight loss research. Transl Behav Med. 2014; 4(3):260–274. doi: 10.1007/s13142-014-0265-0. 25264466 PMC4167891

[9] Collins LM , Murphy SA , Strecher V. The multiphase optimization strategy (MOST) and the sequential multiple assignment randomized trial (SMART): new methods for more potent eHealth interventions. Am J Prev Med. 2007;32(5):S112–S118. 17466815 10.1016/j.amepre.2007.01.022 PMC2062525

[10] Collins LM , Murphy SA , Bierman KL. A conceptual framework for adaptive preventive interventions. Prevent sci. 2004;5(3):185–196. 10.1023/b:prev.0000037641.26017.00 PMC3544191 15470938

[11] Nahum-Shani I , Dziak JJ , Walton MA , Dempsey W. Hybrid experimental designs for intervention development: what, why, and how. Adv Methods Pract Psychol Sci. 2022;5(3):25152459221114279. 10.1177/25152459221114279 PMC10024531 36935844

[12] Collins LM , Nahum-Shani I , Almirall D. Optimization of behavioral dynamic treatment regimens based on the sequential, multiple assignment, randomized trial (SMART). Clin Trials. 2014;11(4):426–434. 24902922 10.1177/1740774514536795 PMC4257903

[13] Bigirumurame T , Uwimpuhwe G , Wason J. Sequential multiple assignment randomized trial studies should report all key components: a systematic review. J Clin Epidemiol, 2022;142:152–160. 34763037 10.1016/j.jclinepi.2021.11.007 PMC7613855

[14] Jaehne A , Loessl B , Frick K , Berner M , Hulse G , Balmford J. The efficacy of stepped care models involving psychosocial treatment of alcohol use disorders and nicotine dependence: a systematic review of the literature. Curr Drug Abuse Rev. 2012;5(1):41–51. 22280331 10.2174/1874473711205010041

[15] Miller CK. Adaptive intervention designs to promote behavioral change in adults: what is the evidence? Curr Diabetes Rep. 2019;19(2):1–9. 10.1007/s11892-019-1127-4 30684109

[16] Murphy SA. An experimental design for the development of adaptive treatment strategies. Stat Med. 2005;24(10):1455–1481. 15586395 10.1002/sim.2022

[17] Roberts H , Sowden A , Petticrew M , Arai L , Rodgers M , Britten N. Guidance on the Conduct of Narrative Synthesis in Systematic Reviews. A Product From the ESRC Methods Programme. Version 1. Lancaster, UK: Lancaster University, 2006.

[18] Patrick ME , Lyden GR , Morrell N , et al. Main outcomes of M-bridge: a sequential multiple assignment randomized trial (SMART) for developing an adaptive preventive intervention for college drinking. J Consult Clin Psych. 2021;89(7):601–614. 10.1037/ccp0000663 PMC8371917 34383533

[19] Almirall D , Nahum-Shani I , Wang L , Kasari C. Experimental Designs for Research on Adaptive Interventions: Singly and Sequentially Randomized Trials. Optimization of Behavioral, Biobehavioral, and Biomedical Interventions. Cham, Switzerland: Springer, 2018:89–120.

[20] Germeroth LJ , Benno MT , Kolko Conlon RP , et al. Trial design and methodology for a non-restricted sequential multiple assignment randomized trial to evaluate combinations of perinatal interventions to optimize women’s health. Contemp Clin Trials. 2019;79:111–121. doi: 10.1016/j.cct.2019.03.002. 30851434 PMC6436999

[21] Belzer ME , MacDonell KK , Ghosh S , et al. Adaptive antiretroviral therapy adherence interventions for youth living with HIV through text message and cell phone support with and without incentives: protocol for a sequential multiple assignment randomized trial (SMART). JMIR Res Protoc. 2018;7(12):e11183. 30573448 10.2196/11183 PMC6320399

[22] Naar S , Parsons JT , Stanton BF. Adolescent trials network for HIV-AIDS scale it up program: protocol for a rational and overview. JMIR Res Protoc. 2019;8(2):e11204. 30707102 10.2196/11204 PMC6376339

[23] Fu SS , Rothman AJ , Vock DM , et al. Program for lung cancer screening and tobacco cessation: study protocol of a sequential, multiple assignment, randomized trial. Contemp Clin Trials. 2017;60:86–95. 28687349 10.1016/j.cct.2017.07.002 PMC5558455

[24] Sherwood NE , Butryn ML , Forman EM , et al. The bestFIT trial: a SMART approach to developing individualized weight loss treatments. Contemp Clin Trials. 2016;47:209–216. 26825020 10.1016/j.cct.2016.01.011 PMC4941634

[25] Borsari B , Tevyaw TOL , Barnett NP , Kahler CW , Monti PM. Stepped care for mandated college students: a pilot study. Am J Addict. 2007;16(2):131–137. 17453615 10.1080/10550490601184498 PMC2714908

[26] Borsari B , Hustad JT , Mastroleo NR , et al. Addressing alcohol use and problems in mandated college students: a randomized clinical trial using stepped care. J Consult Clin Psych. 2012;80(6):1062–1074. 10.1037/a0029902 PMC3514601 22924334

[27] Breslin FC , Sobell MB , Sobell LC , Cunningham JA , Sdao-Jarvie K , Borsoi D. Problem drinkers: evaluation of a stepped-care approach. J Subst Abuse. 1998;10(3):217–232. 10689656 10.1016/s0899-3289(99)00008-5

[28] Lyden GR , Vock DM , Sur A , Morrell N , Lee CM , Patrick ME. Deeply tailored adaptive interventions to reduce college student drinking: a real-world application of Q-learning for SMART studies. Prev Sci. 2022;23(6):1–12. 35543888 10.1007/s11121-022-01371-7 PMC9357163

[29] Patrick ME , Boatman JA , Morrell N , et al. A sequential multiple assignment randomized trial (SMART) protocol for empirically developing an adaptive preventive intervention for college student drinking reduction. Contemp Clin Trials. 2020;96:106089. 32717350 10.1016/j.cct.2020.106089 PMC7494596

[30] McKay JR , Drapkin ML , Van Horn DH , et al. Effect of patient choice in an adaptive sequential randomization trial of treatment for alcohol and cocaine dependence. J Consult Clin Psychol. 2015;83(6):1021–1032. doi: 10.1037/a0039534. 26214544 PMC5823027

[31] Schmitz JM , Stotts AL , Vujanovic AA , et al. A sequential multiple assignment randomized trial for cocaine cessation and relapse prevention: tailoring treatment to the individual. Contemp Clin Trials. 2018;65:109–115. 29287664 10.1016/j.cct.2017.12.015 PMC5803345

[32] Carels RA , Young KM , Coit CB , et al. The failure of therapist assistance and stepped-care to improve weight loss outcomes. Obesity. 2008;16(6):1460–1462. 18356835 10.1038/oby.2008.49

[33] Sherwood NE , Crain AL , Seburg EM , et al. BestFIT sequential multiple assignment randomized trial results: a SMART approach to developing individualized weight loss treatment sequences. Ann Behav Med. 2022;56(3):291–304. 34415011 10.1093/abm/kaab061 PMC8887581

[34] Pfammatter AF , Nahum-Shani I , DeZelar M , et al. SMART: study protocol for a sequential multiple assignment randomized controlled trial to optimize weight loss management. Contemp Clin Trials. 2019;82:36–45. 31129369 10.1016/j.cct.2019.05.007 PMC6624080

[35] Carels RA , Miller JC , Selensky JC , et al. Using an acceptance-based behavioral approach as a supplement to obesity treatment: a stepped-care approach. J Context Behav Sci. 2019;12:98–105.

[36] Edelman EJ , Dziura J , Deng Y , et al. A SMARTTT approach to treating tobacco use disorder in persons with HIV (SMARTTT): rationale and design for a hybrid type 1 effectiveness-implementation study. Contemp Clin Trials. 2021;110:106379. 33794354 10.1016/j.cct.2021.106379 PMC8478961

[37] Fernandez ME , Schlechter CR , Del Fiol G , et al. QuitSMART Utah: an implementation study protocol for a cluster-randomized, multi-level sequential multiple assignment randomized trial to increase reach and impact of tobacco cessation treatment in community health centers. Implement Sci. 2020;15(1):1–13. 32000812 10.1186/s13012-020-0967-2 PMC6993416

[38] Zhao SZ , Weng X , Luk TT , et al. Adaptive interventions to optimise the mobile phone-based smoking cessation support: study protocol for a sequential, multiple assignment, randomised trial (SMART). Trials. 2022;23(1):1–11. 35982468 10.1186/s13063-022-06502-7 PMC9387009

[39] Comins CA , Schwartz SR , Phetlhu DR , et al. Siyaphambili protocol: an evaluation of randomized, nurse-led adaptive HIV treatment interventions for cisgender female sex workers living with HIV in Durban, south Africa. Res Nurs Health. 2019;42(2):107–118. doi: 10.1002/nur.21928. 30644999 PMC6666398

[40] Velloza J , Poovan N , Ndlovu N , et al. Adaptive HIV pre-exposure prophylaxis adherence interventions for young South African women: study protocol for a sequential multiple assignment randomized trial. Plos One. 2022;17(4):e0266665. 35417485 10.1371/journal.pone.0266665 PMC9007385

[41] Gonze BDB , Padovani RDC , Simoes MDS , et al. Use of a smartphone app to increase physical activity levels in insufficiently active adults: feasibility sequential multiple assignment randomized trial (SMART). JMIR Res Protoc. 2020;9(10):e14322. 33094733 10.2196/14322 PMC7647811

[42] Buchholz SW , Wilbur J , Halloway S , et al. Study protocol for a sequential multiple assignment randomized trial (SMART) to improve physical activity in employed women. Contemp Clin Trials. 2020;89:105921. 31899371 10.1016/j.cct.2019.105921 PMC7242143

[43] Simoes MDSMP , de Barros Gonze B , Leite Proenca N , et al. Use of a smartphone app combined with gamification to increase the level of physical activity of adults and older adults: protocol of a sequential multiple assignment randomized trial. Trials. 2019;20(1):780. doi: 10.1186/s13063-019-3879-1. 31881987 PMC6935162

[44] Corbin KD , Igudesman D , Addala A , et al. Design of the advancing care for Type 1 diabetes and obesity network energy metabolism and sequential multiple assignment randomized trial nutrition pilot studies: an integrated approach to develop weight management solutions for individuals with type 1 diabetes. Contemp Clin Trials. 2022;117:106765. 35460915 10.1016/j.cct.2022.106765 PMC9164141

[45] Carels RA , Wott CB , Young KM , et al. Successful weight loss with self-help: a stepped-care approach. J Behav Med. 2009;32(6):503–509. 19521759 10.1007/s10865-009-9221-8 PMC3533369

[46] Liberali R , Del Castanhel F , Kupek E , Assis MAAD. Latent class analysis of lifestyle risk factors and association with overweight and/or obesity in children and adolescents: systematic review. Child Obes. 2021;17(1):2–15. 33306451 10.1089/chi.2020.0115

[47] Bouldin ED , Aikens JE , Piette JD , Trivedi RB. Relationship and communication characteristics associated with agreement between heart failure patients and their carepartners on patient depressive symptoms. Aging Ment Health, 2019;23(9):1122–1129. 30569750 10.1080/13607863.2018.1481923 PMC6586543

[48] Mayberry LS , Greevy RA , Huang L-C , Zhao S , Berg CA. Development of a typology of diabetes-specific family functioning among adults with type 2. Ann Behav Med. 2021;55(10):956–969. 33761527 10.1093/abm/kaab009 PMC8489307

[49] Dansinger ML , Tatsioni A , Wong JB , Chung M , Balk EM. Meta-analysis: the effect of dietary counseling for weight loss. Ann Int Med. 2007;147(1):41–50. 17606960 10.7326/0003-4819-147-1-200707030-00007

[50] Panel DG. Treating Tobacco Use and Dependence: 2008 Update. Rockville, MD: US Department of Health and Human Services, 2008.

